# Same-session dual chromophore riboflavin/UV-A and rose bengal/green light PACK-CXL in *Acanthamoeba* keratitis: a case report

**DOI:** 10.1186/s40662-024-00420-2

**Published:** 2025-01-03

**Authors:** Farhad Hafezi, Jürg Messerli, Emilio A. Torres-Netto, Nan-Ji Lu, M. Enes Aydemir, Nikki L. Hafezi, Mark Hillen

**Affiliations:** 1 ELZA Institute AG, Bahnhofstrasse 15, 8001 Zurich, Switzerland; 2 Laboratory of Ocular Cell Biology, Center for Applied Biotechnology and Molecular Medicine, University of Zurich, Zurich, Switzerland; 3 Faculty of Medicine, University of Geneva, Geneva, Switzerland; 4 USC Roski Eye Institute, University of Southern California, Los Angeles, CA USA; 5 Department of Ophthalmology, University of Wenzhou, Wenzhou, China; 6 Department of Ophthalmology, NYU Grossman School of Medicine, New York, NY USA; 7 Department of Ophthalmology, University Hospital Basel, Basel, Switzerland; 8 Department of Ophthalmology, Paulista School of Medicine, Federal University of Sao Paulo, Sao Paulo, Brazil; 9 School of Medicine and Health Sciences, University of Antwerp, Wilrijk, Belgium

**Keywords:** *Acanthamoeba* keratitis, PACK-CXL, Riboflavin, UV-A, Rose bengal, Green light

## Abstract

**Background:**

*Acanthamoeba* keratitis (AK) is the most challenging corneal infection to treat, with conventional therapies often proving ineffective. While photoactivated chromophore for keratitis-corneal cross-linking (PACK-CXL) with riboflavin/UV-A has shown success in treating bacterial and fungal keratitis, and PACK-CXL with rose bengal/green light has demonstrated promise in fungal keratitis, neither approach has been shown to effectively eradicate AK. This case study explores a novel combined same-session treatment approach using both riboflavin/UV-A and rose bengal/green light in a single procedure.

**Case presentation:**

A 44-year-old patient with active AK in the left cornea, unresponsive to 10 months of conventional treatment according to American Academy of Ophthalmology (AAO) guidelines, was treated using same-session sequential PACK-CXL with riboflavin/UV-A (365 nm) irradiation (10 J/cm^2^) and rose bengal/green light (522 nm) irradiation (5.4 J/cm^2^) in a single setting. The procedure was repeated twice due to persistent signs of inflammation and infection. After three combined same-session PACK-CXL treatments, the patient’s cornea converted to a quiescent scar, and symptoms of ocular pain, photophobia, epiphora, and blepharospasm resolved. Confocal microscopy revealed no detectable *Acanthamoeba* cysts. The patient currently awaits penetrating keratoplasty.

**Conclusions:**

The same-session combination of riboflavin/UV-A and rose bengal/green light PACK-CXL effectively treated a patient with confirmed AK that was resistant to conventional medical therapy, suggesting that using two chromophores in a single procedure may represent a future treatment alternative for AK.

**Supplementary Information:**

The online version contains supplementary material available at 10.1186/s40662-024-00420-2.

## Background

*Acanthamoeba* keratitis (AK) is a rare but severe corneal infection caused by a protozoan parasite. It has a devastating impact on patients’ vision and quality of life and is responsible for up to 5% of all contact lens-associated keratitis cases [1]. AK therapy typically involves months to years of intensive topical treatment with antiseptic agents, often combined with antibiotic and antifungal agents. However, initial treatment failure occurs in 39% of cases, primarily due to the parasite’s cystic form which is highly resistant to conventional antimicrobial and antiseptic agents, and its ability to penetrate deep into the cornea [2].

In 2008, a new treatment modality for infectious keratitis, known as PACK-CXL, was developed, which utilized riboflavin (RF) and UV-A light [3–5]. This approach was effective in treating bacterial and fungal keratitis in the cornea through a direct pathogen-killing effect via oxidative stress [6]. However, RF/UV-A PACK-CXL was not able to successfully eradicate AK [7, 8]. Another chromophore and light combination using rose bengal (RB) and 522 nm green light (RB/green) was then suggested for the treatment of infectious keratitis [9]. RB/green PACK-CXL had produced promising results in treating both bacterial and in particular fungal keratitis [10, 11]. However, neither this approach [12] nor RF/UV-A PACK-CXL [13–17] have proved to be sufficiently effective against AK.

Here, we present a novel combined approach using same-session sequential PACK-CXL with RF/UV-A and RB/green light in the same procedure as an adjunct to standard-of-care antimicrobial therapy, to successfully treat a patient with AK who had previously undergone unsuccessful medical treatment for a year. This novel dual chromophore approach takes advantage of the non-overlapping absorption spectra maxima of both chromophores, allowing them to be combined effectively into a single treatment. The sequential application of both light/chromophore applications in a single treatment may represent a promising alternative for treating AK.

## Case presentation

We conducted a single-center, single-patient case study to evaluate the efficacy of a combined same-session approach using PACK-CXL with sequential RF/UV-A and RB/green light in treating AK.

A 44-year-old patient was referred to our clinic with active AK in the left cornea after extended contact lens wear. Before referral, the AK diagnosis was confirmed using culture from both the cornea and the contact lens case, Giemsa staining, confocal microscopy, and polymerase chain reaction. The patient was then treated unsuccessfully for a total of 10 months according to the American Academy of Ophthalmology guidelines [18] without resolution or clinical improvement in corneal findings before referral. Specifically, the initial therapy consisted of hexamidine diisethionate 0.1% eyedrops (Desomedin DD, Bausch + Lomb, Zug, Switzerland) four times daily, chlorhexidine 0.002% every 2 h (q2h), propamidine isethionate 0.1% q2h and polyhexamethylenebiguanide (PHMB) 0.02% (all prepared by the referring hospital’s compound pharmacy).

Furthermore, the patient received oral valaciclovir (Valtrex 500 mg, GlaxoSmithKline, Münchenbuchsee, Switzerland) twice daily, nightly topical acyclovir ointment (Acivision Augensalbe 30 mg/g, OmniVision AG, Neuhausen am Rheinfall, Switzerland), and topical 1% voriconazole drops (Pfizer AG, Zurich, Switzerland) five times daily. For full details, please refer to Appendix A, Supplemental Table 1. Written informed consent was obtained regarding the publication of the case.

### Diagnostic assessment

**Fig. 1:**
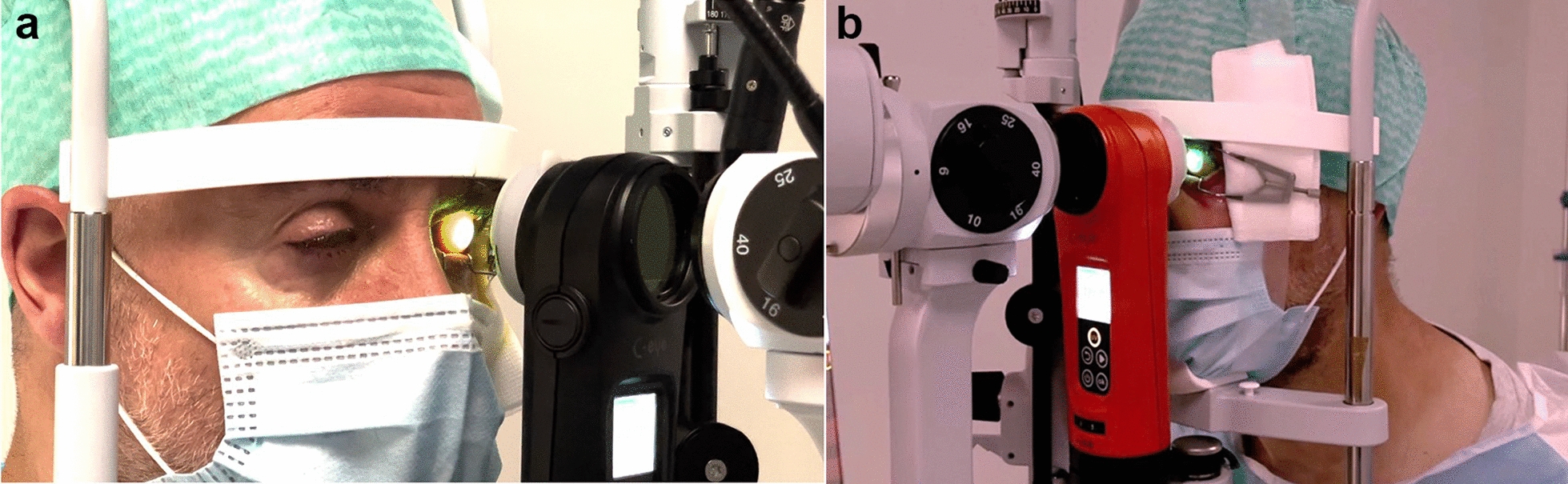
Same-session photoactivated chromophore for keratitis-corneal cross-linking (PACK-CXL) treatment using two chromophores. **a** Irradiation with UV-A using a commercially available CXL device; **b** Irradiation with green light using a custom-built irradiation device

Upon presentation, the patient exhibited intense ocular pain, excessive epiphora, photophobia, and blepharospasm in the left eye. Corrected distance visual acuity (CDVA) was 20/200. Slit-lamp examination revealed diffuse hyperemia of the conjunctiva, and the cornea presented with diffuse full-thickness infiltrates in the absence of a ring infiltrate. The remainder of the slit-lamp examination was normal (Fig. 1a). *Acanthamoeba* cysts were identified in the fluid from the contact lens container via polymerase chain reaction. Confocal microscopy showed *Acanthamoeba* cysts in the superficial and deep corneal stroma.

### Intervention

**Fig. 2:**
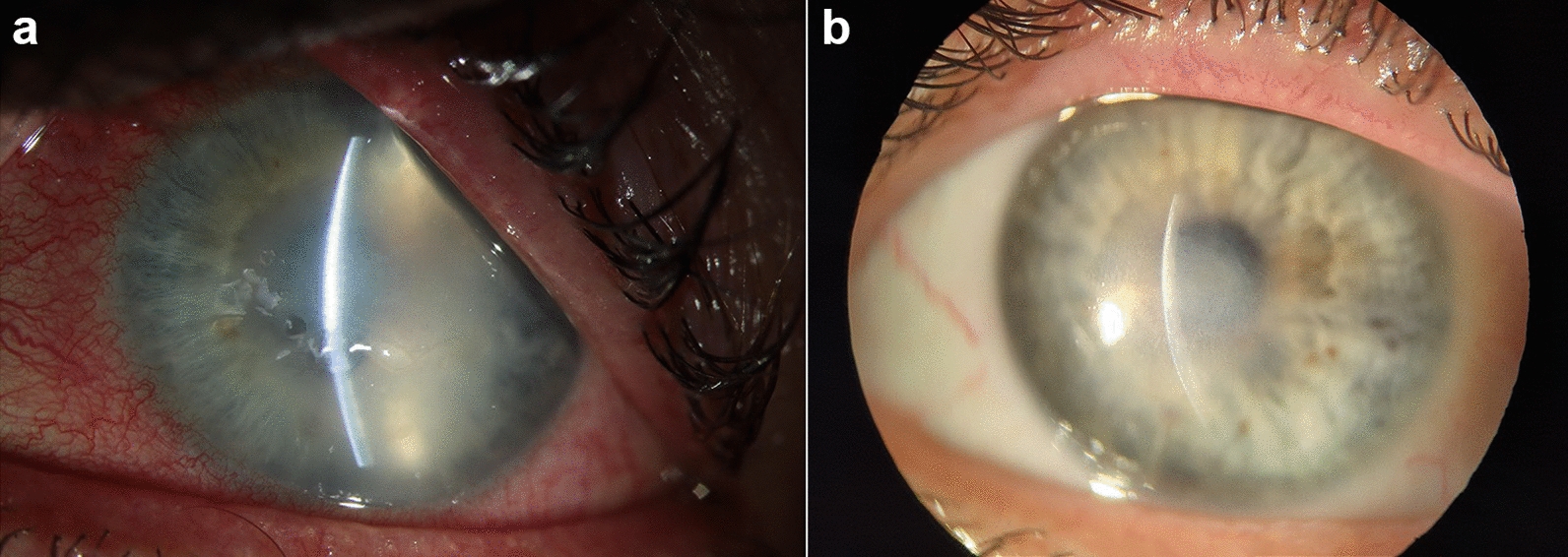
Photograph of the cornea before and after the same-session dual chromophore photoactivated chromophore for keratitis-corneal cross-linking (PACK-CXL) treatment. **a** Prior to initiation of the PACK-CXL treatment, the cornea presented with all signs of acute *Acanthamoeba* keratitis; **b** Six months after the third same-session PACK-CXL treatment, the cornea displays a quiescent deep stromal scar

Following national legal guidelines for compassionate use and a detailed informed consent signed by the patient, we performed the initial combined PACK-CXL treatment on June 14, 2021. In brief, following a 9-mm epithelial layer abrasion, 0.1% RF solution (Ribo-Ker, EMAGine, Zug, Switzerland) every 2 min for 20 min, followed by instillation of 0.1% RB solution (Grosse Apotheke Bichsel, Interlaken, Switzerland) every 2 min for 20 min. The chromophores were instilled onto the surface of the cornea, ensuring the site of infection became saturated. After rinsing off the excess chromophore from the corneal surface with balanced salt solution, the cornea was irradiated with 365 nm UV-A light at 18 mW/cm^2^ for 9 min and 15 s (C-eye device, EMAGine, Zug, Switzerland), corresponding to a total fluence of 10 J/cm^2^. UV-A irradiation was immediately followed by irradiation with 522 nm green light at 15 mW/cm^2^ for 6 min, corresponding to a total fluence of 5.4 J/cm^2^ (Fig. 2).

We reperformed the combined procedure twice, at 4 weeks (July 15) and 16 weeks (October 4, 2021) after the initial procedure. Each PACK-CXL treatment comprised of same-session sequential RF/UV-A (365 nm) irradiation with 10 J/cm^2^ (C-eye and Ribo-Ker, EMAGine, Switzerland) and RB/green light (522 nm) irradiation (custom-built device, 0.1% RB) with 5.4 J/cm^2^ in a single setting.

### Follow-up and outcome assessment

Despite a reduction in AK activity and an improvement in the patient’s symptoms in the 4 weeks following the first combined PACK-CXL procedure, signs of active infection persisted. The initial therapy was therefore modified to hexamidine diisethionate eyedrops three times daily and dexamethasone sodium phosphate eyedrops (DexaFree, Théa Pharma, Switzerland) twice daily.

A second combined same-session RF/UV-A & RB/green light treatment with similar irradiation settings to the primary procedure was performed on July 15, 2021. Clinical signs of active infection improved further, however, corneal edema and infiltrates remained visible in the deep stroma, and medication remained unchanged.

**Fig. 3:**
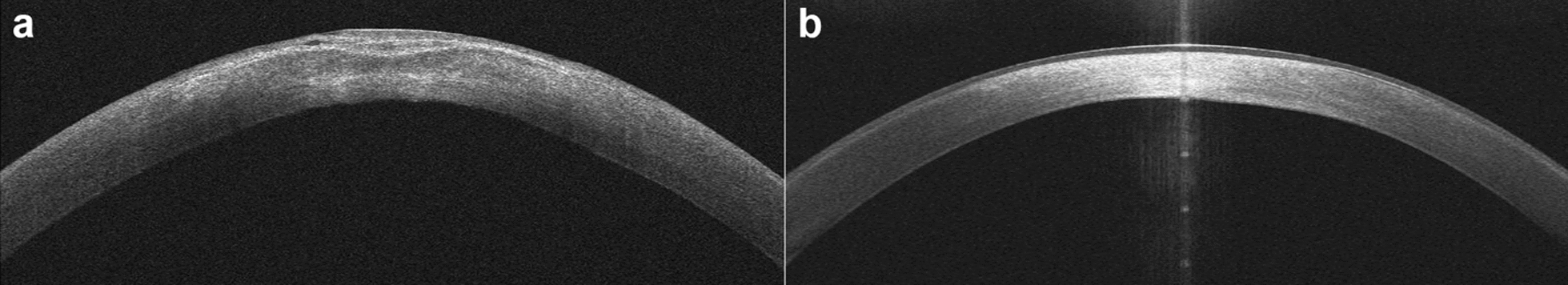
Anterior segment-optical coherence tomography. **a** Before initiation of the photoactivated chromophore for keratitis-corneal cross-linking (PACK-CXL) therapy, the cornea showed marked edema; **b** Six months after the third same-session PACK-CXL treatment, the central cornea shows a full-thickness scar and epithelial remodeling

A third combined procedure was performed on October 4, 2021, again using the same technical irradiation settings. Following the third combined same-session procedure, the cornea showed resolution of edema (Fig. 3) and a steady decrease in infiltrate size over the next 6 months and presented as a quiescent scar in April 2022. Medication was tapered out between November 2021 and April 2022. The patient’s previous symptoms of ocular pain, photophobia, epiphora, and blepharospasm had resolved (Fig. 1b). The patient’s CDVA improved to 20/100, and confocal microscopy was unable to detect *Acanthamoeba* cysts.

### Corneal healing

Slit-lamp biomicroscopic and corneal topography assessments demonstrated a progressive reduction in corneal infiltration, epithelial defects, and stromal haze throughout the follow-up period. By the end of the 18-month follow-up, the cornea appeared clear and stable, with no signs of residual AK or complications.

### Confocal microscopy

Confocal microscopy examination in April 2022 did not reveal any detectable *Acanthamoeba* cysts in the corneal stroma. The patient is currently awaiting a penetrating keratoplasty to restore visual function further. These findings suggest that the combined approach of sequential RF/UV-A and RB/green light PACK-CXL was effective in treating this case of confirmed AK, which had been resistant to conventional medical treatment for 10 months prior to our intervention.

## Discussion

The successful treatment of this patient with confirmed AK using a combined same-session approach of RF/UV-A and RB/green light PACK-CXL presents a potential alternative treatment modality for AK, which may be of particular value in late-presenting cases, severe cases, and in cases where cysts are present very deep in the stroma. There is an unmet need for interventions that can speed the time to resolution of AK ulcers; a recent retrospective case review study spanning 25 years of follow-up [19] found that the overall healing time of patients with AK was 12.5 ± 3.5 months, while patients with more severe (stage III) corneal ulcers had significantly longer healing times (16.2 ± 3.7 months).

**Fig. 4:**
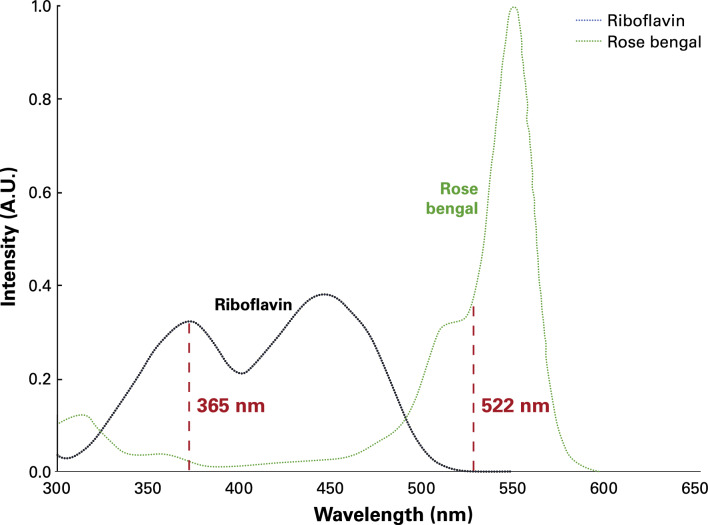
Absorption spectra of riboflavin and rose bengal showing no competition for energy at the wavelengths used for the UV-A-induced (365 nm) and the green light-induced photoactivated chromophore for keratitis-corneal cross-linking (PACK-CXL, 522 nm)

As neither RF/UV-A nor RB/green light PACK-CXL has been effective individually in treating AK [12–17], the combination of the two chromophores and their distinct photochemical properties may have contributed to the successful outcome in this case. When plotting the absorption spectra of RF and RB, it becomes evident that the chromophores do not compete for the energy of the light at the wavelengths used (Fig. 4). In other words: a cornea can be saturated with both chromophores without one chromophore interfering with the photoactivation of the other. Since RF penetrates deeper into the corneal stroma than RB, we decided to irradiate with UV-A first, followed by 522 nm green light illumination.

The mechanisms behind the observed synergy of these two photoactivated chromophores remain to be determined. It is possible that the combination treatment may result in a more effective disruption of the amoebic cyst wall, enhancing the penetration of subsequent antimicrobial agents and ultimately leading to cyst destruction. Furthermore, the combined PACK-CXL also induces an increased resistance to digestion, thereby reinforcing the corneal stroma and preventing further tissue degradation through pathogen-produced proteases [20, 21]. Similar to the treatment of fungal keratitis using RB and green light, several sessions were needed to eradicate the *Acanthamoeba* cysts [10, 11].

## Limitations

This case report has some limitations, including the absence of a control group and the potential influence of previous treatments. However, the patient’s history of unsuccessful treatments and significant clinical improvement following the combined PACK-CXL approach using two chromophores suggest that this novel combined treatment played a crucial role in the resolution of the AK infection.

## Conclusion

The repeated combined same-session dual chromophore treatment for AK has demonstrated the utility of a combined RF/UV-A and RB/green light PACK-CXL approach appears to have been successful in treating a patient with confirmed AK, which was resistant to conventional medical treatment before our intervention. Further research, including laboratory studies, larger-scale clinical trials, and trials with long-term follow-up periods are needed to confirm the safety and efficacy of this combined treatment approach and to optimize treatment parameters. Nevertheless, this novel treatment employing two chromophores in a single procedure may represent a future alternative for managing AK.

## Supplementary Information

Supplementary Material 1.

## Data Availability

Data are available on reasonable request. Please contact FH at fhafezi@elza-institute.com.
