# Supplementary material for: Same-session dual chromophore riboflavin/UV-A and rose bengal/green light PACK-CXL in Acanthamoeba keratitis: a case report
Source: Eye Vis (Lond). 2025 Jan 3;12:2. doi: 10.1186/s40662-024-00420-2 (PMC11697719; doi:10.1186/s40662-024-00420-2)
Supplement: Supplementary file 1 — Supplementary Material 1. [file 40662_2024_420_MOESM1_ESM.docx]

**APPENDIX A, SUPPLEMENTAL**

**TABLE 1. Medication regimens.**

| **Date** | **PACK-CXL with riboflavin and rose bengal: medication** |
| --- | --- |
| **10.05.2021** | Valaciclovir tablets, Aciclovir ointment, Vorinocazol drops 5x daily, Hexamidin diisethionat (Desomedin) drops 4x tgl |
| **14.06.2021** | **CXL** |
| **15.06.2021** | Moxifloxacin (Vigamox) drops 2x, Vorinocazol drops 4x, Hexamidin diisethionat (Desomedin) drops |
| **21.06.2021** | Ofloxacin (Floxal) ointment, Moxifloxacin (Vigamox) drops, Hexamidin diisethionat (Desomedin) drops |
| **25.06.2021** | Hexamidin diisethionat (Desomedin) drops, Dexamethason dihydrogenphosphat Dinatrium (DexaFree) drops 2x daily |
| **30.06.2021** | Hexamidin diisethionat (Desomedin) drops 3x, Dexamethason dihydrogenphosphat Dinatrium (DexaFree) drops 2x daily |
| **13.07.2021** | **Re-CXL** |
| **16.07.2021** | Moxifloxacin (Vigamox) drops, Ofloxacin (Floxal) drops, Hexamidin diisethionat (Desomedin) drops |
| **23.07.2021** | Loteprednol etabonate (Lotemax) drops 2x, Hexamidin diisethionat (Desomedin) drops |
| **13.08.2021** | Hexamidin diisethionat (Desomedin) drops 4x, Moxifloxacin (Vigamox) drops 3x daily, Dexamethason dihydrogenphosphat Dinatrium (DexaFree) drops 2x daily |
| **03.09.2021** | Hexamidin diisethionat (Desomedin) drops, Dexamethason dihydrogenphosphat Dinatrium (DexaFree) drops, Moxifloxacin (Vigamox) drops |
| **04.10.2021** | **Re-CXL** |
| **08.10.2021** | Tobramycin (Tobradex) drops, Moxifloxacin (Vigamox) drops, Hexamidin diisethionat (Desomedin) drops |
| **12.10.2021** | Moxifloxacin (Vigamox) drops 4x, Hexamidin diisethionat (Desomedin) drops, Dexamethason dihydrogenphosphat Dinatrium (DexaFree) drops 4x daily |
| **15.10.2021** | Hexamidin diisethionat (Desomedin) drops 3x daily, Dexamethason dihydrogenphosphat Dinatrium (DexaFree) drops 4x daily |
| **10.11.2021** | Hexamidin diisethionat (Desomedin) drops 2x daily, Dexamethason dihydrogenphosphat Dinatrium (DexaFree) drops 2x daily |
| **16.12.2021** | Hexamidin diisethionat (Desomedin) drops 2x daily, Dexamethason dihydrogenphosphat Dinatrium (DexaFree) drops 2x daily |
| **02.02.2022** | Stop all medication |
| **28.11.2022** | Hexamidin diisethionat (Desomedin) drops 2x daily, Dexamethason dihydrogenphosphat Dinatrium (DexaFree) drops 2x daily, Moxifloxacin (Vigamox) drops 2x |
